# From transcriptome to biological function: environmental stress in an ectothermic vertebrate, the coral reef fish *Pomacentrus moluccensis*

**DOI:** 10.1186/1471-2164-8-358

**Published:** 2007-10-05

**Authors:** Karin S Kassahn, Ross H Crozier, Alister C Ward, Glenn Stone, M Julian Caley

**Affiliations:** 1 School of Marine and Tropical Biology, James Cook University, Townsville, QLD 4811, Australia; 2 School of Medicine, Deakin University, Geelong, VIC 3217, Australia; 3 CSIRO Mathematical and Information Sciences, North Ryde, NSW 2113, Australia; 4 Australian Institute of Marine Sciences, PMB No.3, Townsville, QLD 4810, Australia; 5 ARC Centre of Excellence in Bioinformatics and Institute for Molecular Bioscience, The University of Queensland, Brisbane, QLD 4072, Australia

## Abstract

**Background:**

Our understanding of the importance of transcriptional regulation for biological function is continuously improving. We still know, however, comparatively little about how environmentally induced stress affects gene expression in vertebrates, and the consistency of transcriptional stress responses to different types of environmental stress. In this study, we used a multi-stressor approach to identify components of a common stress response as well as components unique to different types of environmental stress. We exposed individuals of the coral reef fish *Pomacentrus moluccensis *to hypoxic, hyposmotic, cold and heat shock and measured the responses of approximately 16,000 genes in liver. We also compared winter and summer responses to heat shock to examine the capacity for such responses to vary with acclimation to different ambient temperatures.

**Results:**

We identified a series of gene functions that were involved in all stress responses examined here, suggesting some common effects of stress on biological function. These common responses were achieved by the regulation of largely independent sets of genes; the responses of individual genes varied greatly across different stress types. In response to heat exposure over five days, a total of 324 gene loci were differentially expressed. Many heat-responsive genes had functions associated with protein turnover, metabolism, and the response to oxidative stress. We were also able to identify groups of co-regulated genes, the genes within which shared similar functions.

**Conclusion:**

This is the first environmental genomic study to measure gene regulation in response to different environmental stressors in a natural population of a warm-adapted ectothermic vertebrate. We have shown that different types of environmental stress induce expression changes in genes with similar gene functions, but that the responses of individual genes vary between stress types. The functions of heat-responsive genes suggest that prolonged heat exposure leads to oxidative stress and protein damage, a challenge of the immune system, and the re-allocation of energy sources. This study hence offers insight into the effects of environmental stress on biological function and sheds light on the expected sensitivity of coral reef fishes to elevated temperatures in the future.

## Background

Microarray technology provides a powerful tool for investigating gene regulation and its significance for biological function. However, our understanding of such relationships during environmental stress remains fragmentary, especially in vertebrates. In particular, the commonality, or otherwise, of the responses of vertebrates to different environmental stresses remain poorly understood. Recently, some understanding of responses to individual stresses, in particular those related to thermal stress in teleost fishes, has been gained. In these species, thermal stress can lead to changes in ventilation and circulation rates [1], changes in mitochondrial densities and their properties [2,3], and a reduction in cellular oxygen levels [4,5]. Reduced cellular oxygen levels can lead to increased levels of oxidative stress and hence, the cellular response to thermal stress often includes responses aimed at alleviating oxidative stress [2,6]. For example, antioxidant enzymes, such as superoxide dismutase, catalase, and glutathione peroxidase are commonly activated during thermal stress [7,8]. Oxidative stress, and the cellular damage associated with it, can in turn induce a heat shock response primarily aimed at the molecular repair of protein damage [9-12]. In addition, thermal stress can lead to extensive changes in gene expression [13-16]. These transcriptional responses are likely the result of the stress-dependent activation of only a limited number of upstream regulators, namely the activation mitogen-activated protein kinases (MAPK), in particular the JNK and p38 signalling pathways [17-19], and immediate early genes [20,21]. These complex transcriptional responses to stress necessarily precede adjustments at the protein level, and thus, are expected to form an important component of the cellular response to stress.

To advance our current limited understanding of the consistency of transcriptional stress responses across different environmental stress types, it is necessary to examine stress responses to a diversity of stressors simultaneously and a suitable model organism is required. Teleost fishes, and coral reef fishes in particular, are one such potential model. Being ectothermic and aquatic and because their physiology is tightly linked to the environment, teleost fishes are particularly amenable to experimental manipulation of environmental stresses and have hence become important models for environmental stress studies [13-16]. Coral reef fishes inhabit relatively stable environments compared to those experienced by many other teleost fishes, and are so likely to have increased sensitivity towards thermal and environmental anomalies. By identifying the most sensitive biological processes in organisms with limited tolerance towards environmental anomalies, we gain understanding of the types of processes that must have been the targets of adaptation in organisms that exhibit broad environmental tolerances [see [2,22] for examples]. In this way, studying environmental stress responses in coral reef fishes may provide fundamental insights into mechanisms of environmental adaptation.

While there are currently no species-specific microarrays available for any species of coral reef fish, we have previously shown that the Compugen 16 K oligonucleotide microarray developed for the zebrafish, *Danio rerio*, can be successfully used to study gene responses in the coral reef fish *Pomacentrus moluccensis *[23]. In this previous study, comparative genomic hybridisation experiments showed good cross-hybridisation between the two fish species for most genes represented on the microarray, and differential expression of a set of candidate genes were confirmed by quantitative real-time PCR [23]. Here, we use this previously validated heterologous microarray approach to measure transcriptional responses of the coral reef fish *Pomacentrus moluccensis *to different environmental stressors, including hypoxia, hyposmotic stress, cold, and heat. We also compare heat stress responses of winter- and summer-acclimated fish to estimate seasonal effects on gene regulation. The duration of environmental stress treatments was varied, permitting different aspects of the biological response to be examined. Short-term exposure experiments over three hours investigated early gene responses and upstream regulators of transcriptional stress responses, while prolonged exposure to heat over five days investigated medium-term effects of heat stress. By grouping genes into functional categories and identifying those functional categories most commonly associated with gene responses to different types of environmental stress, we identify components of a common stress response in *P. moluccensis *as well as features unique to different types of environmental stress. We use these gene function responses to infer stress-induced changes in biological and physiological function and discuss the types of biological functions affected by exposure to environmental stress.

## Results

### Early gene responses to different environmental stresses

**Figure 1:**
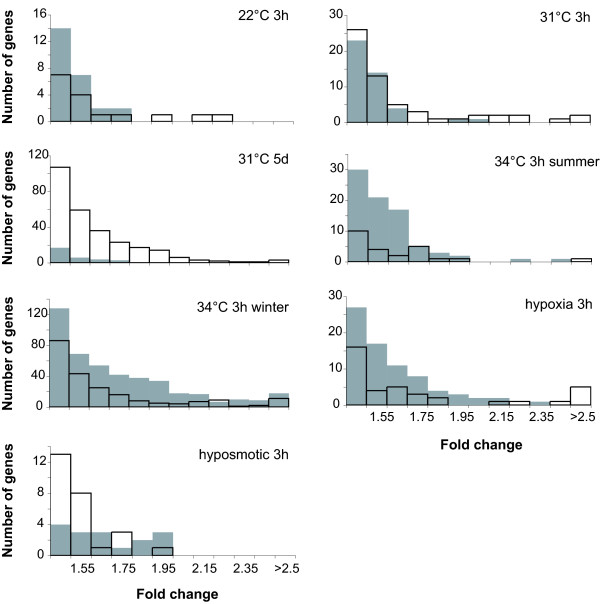
**Summary of gene expression fold changes of *Pomacentrus moluccensis *in response to environmental stresses**. Grey boxes indicate down-regulation, white boxes indicate up-regulation of gene in stressed fish relative to fish kept at ambient conditions.

To characterise environmental stress responses, we analysed the gene expression profiles of 118 individuals. These individuals were from a natural population of *P. moluccensis *and were either subjected to one of the environmental stress treatments or kept at ambient conditions. The microarray platform, data series, and raw data including the .tif image and .spot files are available from the Gene Expression Omnibus website [24] under accessions GPL3365, GSE7499, and GSM181765-GSM181823. Most genes that showed significant expression changes in response to short-term cold, heat, hypoxic or hyposmotic shock were down-regulated compared to the expression levels measured in fish kept at ambient conditions (Figure 1, see Additional File 1 for a complete list of gene responses). Short-term heat shock at 34°C in summer led to significant expression changes at six gene loci, amongst which were *pdip5*, *rhoA*, and *ZCCHC11*, and three genes showed greater than two-fold expression changes (Additional File 1). In contrast to the summer response, the short-term 34°C heat shock response in winter involved many more genes, and the observed expression changes generally were of greater magnitude (Figure 1, see also [23]). In total, 111 gene loci were differentially expressed in the winter-response to short-term heat shock at 34°C. All but six of these genes were down-regulated in heat-stressed fish with fold changes up to 3.8. The early transcriptional response to hypoxia involved ten differentially expressed genes, amongst which were *acin1a*, *β-actin*, *lbr*, and *ela2*, and a total of thirteen genes with greater than two-fold expression changes (Additional File 1). Hyposmotic shock resulted in no significant expression changes, and none of the genes assayed showed greater than two-fold expression changes. It is possible that other tissues, for example gills, might have shown a greater response to hyposmotic challenge, but measurement of expression changes in multiple tissues was outside the scope of this study. Due to this lack of response in liver tissue, hyposmotic stress is not discussed further.

### Gene regulation in response to prolonged heat

**Figure 2:**
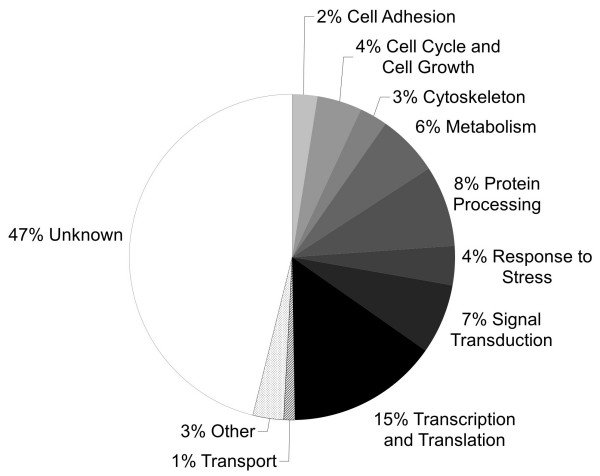
**Gene function categories associated with prolonged heat exposure**. Gene function categories summarise the gene expression response of *Pomacentrus moluccensis *to heat exposure at 31°C over five days.

Prolonged exposure to 31°C over five days resulted in 324 differentially expressed genes, most of which – in contrast to the short-term exposure experiments – were induced and 15 of which showed greater than two-fold expression changes (Figure 1, see Additional File 2 for a complete list of gene responses). Genes encoding protein kinase C substrate 80K-H, rab escort protein 1, semaphorin 3ab, distal-less homeobox 3, and TTF-I interacting peptide 5 showed the greatest regulation (Additional File 2). Differentially expressed genes belonged to a variety of gene functions, amongst which were protein processing, signal transduction, and transcription and translation. Only four percent had been previously associated with the response to stress, while 47 percent were of yet unknown gene function (Figure 2).

### Common effects of stress on biological function

The gene expression responses to different environmental stressors can be tested for the presence of common gene function responses. Such an approach can aid the interpretation of gene regulation in terms of the types of biological functions that are affected. We thus grouped individual genes on the basis of their gene function. We then tested whether genes of a certain function were more likely to be differentially expressed in response to environmental stress than expected if differential expression was independent of gene function. Such gene set analysis revealed significant association of a number of gene functional groups with the gene responses to different types of environmental stress (Table 1). In particular, gene functions related to cell growth and cytoskeleton, protein turnover, and metabolism were consistently associated with gene responses to environmental stress, regardless of the type of stressor applied (Table 1).

**Table 1:** Results of gene set analysis of gene expression responses to environmental stress in *Pomacentrus moluccensis*

**Gene Ontology/GO ID**	**# of probes on array**	**# of genes on array**	**p-value (FDR-corrected)**						
			
			**31°C 5 d**	**22°C 3 h**	**31°C 3 h**	**34°C 3 h sum.**	**34°C 3 h winter**	**Hypoxia**	**Hyposm.**
**Cell growth and Cytoskeleton**									
Actin cytoskeleton organization/GO:0030036	253	81	**0.019**	**0.000**	**0.000**	**0.000**	**0.000**	**0.000**	**0.013**
Anti-apoptosis/GO:0006916	76	72	**0.017**	**0.000**	**0.079**	**0.033**	**0.025**	**0.000**	**0.065**
Induction of apoptosis/GO:0006917	75	73	**0.001**	**0.001**	**0.034**	**0.007**	**0.002**	0.220	**0.000**
Cell growth/GO:0016049	65	63	**0.044**	**0.000**	0.454	**0.000**	**0.001**	**0.075**	0.167
Mitotic cell cycle/GO:0000278	30	30	0.106	**0.082**	0.572	0.127	**0.027**	**0.069**	0.417
**Protein turnover**									
Regulation of translation/GO:0006445	82	81	**0.000**	**0.000**	**0.002**	**0.005**	**0.000**	**0.001**	**0.012**
Ubiquitin-dependent protein catabolism/GO:0006511	98	96	0.200	**0.092**	**0.024**	**0.002**	**0.001**	**0.003**	**0.011**
Protein folding/GO:0006457	75	74	**0.000**	0.191	**0.005**	**0.000**	**0.002**	**0.000**	0.446
**Metabolism**									
Carbohydrate metabolism/GO:0005975	67	67	**0.057**	0.217	**0.076**	**0.011**	**0.000**	**0.001**	**0.088**
Lipid metabolism/GO:0006629	75	75	**0.001**	0.113	**0.007**	**0.000**	**0.000**	0.127	**0.018**
Glycolysis/GO:0006096	31	31	0.319	0.598	0.304	0.127	**0.009**	**0.086**	0.226
Gluconeogenesis/GO:0006094	21	21	0.644	0.408	0.353	0.154	**0.013**	**0.087**	0.430
**Stress**									
Response to pest, pathogen or parasite/GO:0009613	92	89	**0.000**	**0.000**	**0.000**	**0.000**	**0.000**	**0.000**	**0.024**
Response to temperature stimulus/GO:0009266	57	52	**0.023**	0.148	**0.001**	**0.000**	**0.010**	0.163	0.225
Osmoregulation GO:0018987	16	16	0.272	0.136	0.162	**0.035**	**0.067**	0.457	0.178
Response to oxidative stress/GO:0006979	22	22	**0.072**	0.306	0.274	0.270	0.865	0.273	0.239
**Other**									
DNA repair/GO:0006281	84	84	0.115	**0.040**	0.291	**0.081**	**0.000**	**0.068**	0.365
Hemoglobin complex/GO:0005833	16	11	**0.017**	0.651	0.227	**0.027**	**0.050**	0.151	0.446

For each gene ontology an aggregate score was computed based on the p-values for differential expression of the genes in that category. The significance of the resulting gene set score was determined by random sampling of the data using the software ermineJ [72]. P-values are FDR-corrected with values smaller than 0.1 indicated in bold. "3 h" indicates three hour exposure,"5 d" five day exposure to stressor, 'sum." refers to exposure experiments performed in summer, "Hyposm." refers to hyposmotic exposure experiments.

### Variable gene responses across stress treatments

**Figure 3:**
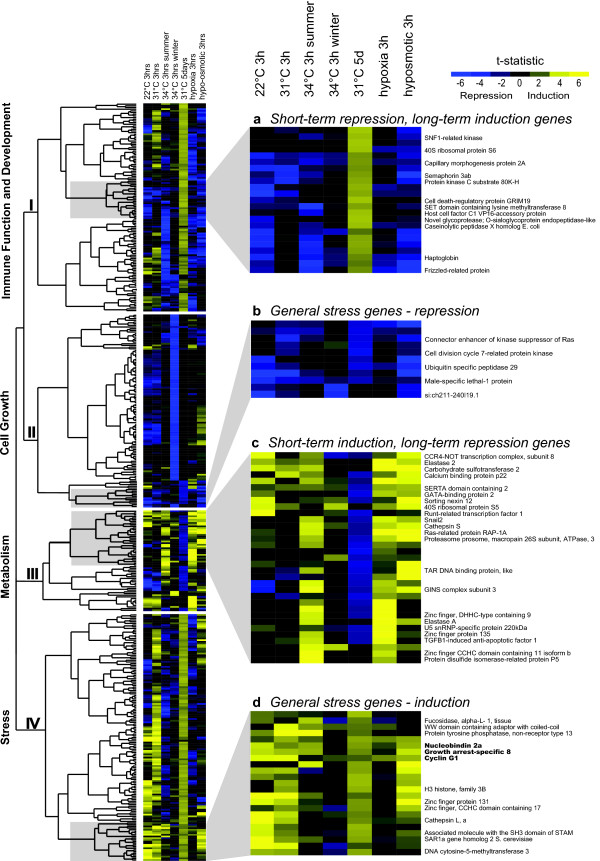
**Hierarchical clustering of genes showing significant gene regulation in response to environmental stress**. The results of hierarchical clustering of genes that showed differential expression in response to different types of environmental stress in *Pomacentrus moluccensis *are shown in form of a heat map. Values presented are the values of the t-statistic summarising the expression responses across biological replicates. Where spots on the microarray could be unambiguously annotated with gene names, these are given to the right of the corresponding row in the heat map. Three genes that showed the most consistent induction across stress treatments are in bold. "3 h" indicates three hour exposure, "5 d" five day exposure to stressor.

Having identified common gene function responses, we wanted to visualise the responses of individual genes across stress treatments and test for the presence of a set of commonly induced, or suppressed, genes. For this purpose, we extracted the expression data from all differentially expressed genes across all treatments and organised the expression profiles using unsupervised hierarchical clustering. Hierarchical clustering identified four main clusters, with genes belonging to the same cluster showing coordinated responses across stressors (Figure 3). While we identified groups of co-regulated genes, gene responses varied considerably across stressors. Individual genes that showed differential expression in one treatment did not necessarily show a significant response in another treatment, and some genes showed expression changes in opposite directions in response to different stressors (Figure 3). Cluster I was comprised of genes which were strongly induced in the 31°C five days treatment, but showed variable responses in the short-term treatments. A subset of cluster I genes was generally down-regulated in the short-term treatments (group a, Figure 3). In order to determine whether there was a relationship between cluster identity and gene function, i.e. whether co-regulated genes shared similar gene functions, we performed gene class testing on the clusters identified by hierarchical clustering using over-representation analysis. The gene ontology 'MHC class I protein binding' was significantly over-represented amongst cluster I genes (Table 2). Cluster II contained a series of genes that were strongly down-regulated in the 34°C winter treatment and which showed variable expression responses in the other treatments. A subset of cluster II genes was down-regulated in most stress treatments examined (group b, Figure 3). Cluster II genes showed significant over-representation of gene functions related to cell growth and cytoskeleton (Table 2). Cluster III was comprised of genes that were strongly suppressed during prolonged heat at 31°C and most of which were induced in the short-term treatments, especially group c genes (Figure 3). Metabolic gene functions were significantly over-represented amongst cluster III genes (Table 2). Lastly, cluster IV included genes that were strongly induced in the 31°C five days treatment and which were generally also induced in the short-term treatments, especially genes in group d (Figure 3). Stress-related gene functions showed a trend for over-representation amongst cluster IV genes, but just exceeded the threshold for significance after FDR-correction (Table 2).

**Table 2:** Results of over-representation analysis of gene ontologies

**Gene Ontology**	**GO ID**	**Number of probes on array**	**Number of genes on array**	**Number of genes in gene cluster**	**Raw p-value**	**FDR-corrected p-value**
**Cluster I-Immune Function and Development**						
MHC class I protein binding	GO:0042288	7	7	2	0.0000	0.0561
Neurite morphogenesis	GO:0048812	62	60	3	0.0032	0.1289
Cell fate determination	GO:0001709	59	45	2	0.0106	0.1807
**Cluster II-Cell Growth**						
Vesicle transport along actin filament	GO:0030050	181	9	2	0.0000	0.0251
SWR1 complex	GO:0000812	182	10	2	0.0000	0.0119
Cortical actin cytoskeleton	GO:0030864	183	11	2	0.0000	0.0122
Condensed nuclear chromosome	GO:0000794	13	13	2	0.0001	0.0168
Histone acetylation	GO:0016573	186	14	2	0.0001	0.0178
Actin filament	GO:0005884	187	15	2	0.0001	0.0190
Meiosis	GO:0007126	51	51	3	0.0002	0.0269
M phase of mitotic cell cycle	GO:0000087	97	96	3	0.0024	0.0779
**Cluster III-Metabolism**						
Sterol metabolism	GO:0016125	12	12	3	0.0000	0.0001
Trypsin activity	GO:0004295	14	14	3	0.0000	0.0001
Positive regulation of protein metab.	GO:0051247	18	18	3	0.0000	0.0002
Cholesterol metabolism	GO:0008203	18	18	3	0.0000	0.0002
**Cluster IV-Stress**						
Lytic vacuole	GO:0000323	42	42	3	0.0001	0.2378
Lysosome	GO:0005764	54	54	3	0.0004	0.3168
Response to UV	GO:0009411	6	5	1	0.0004	0.1620
Replicative cell aging	GO:0001302	5	5	1	0.0004	0.1296
Response to water deprivation	GO:0009414	5	5	1	0.0004	0.1080
Response to reactive oxygen species	GO:0000302	7	7	1	0.0008	0.1349

We tested for significant over-representation of gene ontologies of genes within a cluster compared to the total complement of genes represented on the microarray. Gene clusters were identified by hierarchical clustering of significant gene responses across stressors (see Figure 3 for cluster identification)

## Discussion

This is the first study to examine the effects of different types and durations of environmental stress on transcriptional regulation in an ectothermic vertebrate, in this case the coral reef fish *P. moluccensis*. We identified a series of gene functions that were consistently associated with gene responses to environmental stress, suggesting common effects of different types of stress on biological function. However, these common responses were achieved by the regulation of largely independent sets of genes. Thus, there was conservation of gene function responses, but variability in the responses of individual genes. While the expression response of individual genes varied depending on the types and durations of stresses applied, we have identified groups of co-regulated genes, the genes within which shared similar gene functions.

### Common gene function responses elucidate how stress affects *P. moluccensis*

Some gene functions were consistently associated with the stresses applied here, and thus are likely to reflect the common effects of environmental stress on biological function in this species. Consistent with the observation that stress negatively affects cell growth [25,26] and that, therefore, actin cytoskeletal elements are commonly regulated in response to stress [27,28], the gene ontologies 'actin cytoskeleton organization and biogenesis', 'anti-apoptosis', 'induction of apoptosis', and 'cell cycle' were associated with most stress responses measured here. Individual stress-responsive genes in this category included *β-actin*, *plectin 1*, *dynein*, *spectrin*, *tubulin beta-2*, *filamin 2*, *acin1a*, *GRIM19*, *TP53INP1*, *septin 3*, *cyclin G1*, *cdca3*, and *bax *(Additional Files 1 and 2). Three genes encoding for nucleobindin 2a, growth arrest-specific 8, and cyclin G1 and associated with cell cycle and cell growth, showed the most consistent and significant induction across all stress treatments examined (Figure 3), making them good candidates for molecular stress biomarkers in *P. moluccensis*.

Gene functions related to protein turnover, such as 'regulation of translation', 'protein folding', and 'ubiquitin-dependent protein catabolism', were also commonly associated with the gene responses of *P. moluccensis *to stress. Individual genes that contributed to this component of the common stress response included *pdip5*, *USP29*, *psmc3*, *fbxl12*, the translation initiation factors *eif2b2*, *eif2c1*, *eif4b*, and the ribosomal proteins *rps6*, *mrpl30*, *mrpl36*. Stress can increase rates of protein damage and ubiquitin-dependent protein catabolism [19,29,30]. Association of the gene functions 'protein folding' and 'ubiquitin-dependent protein catabolism' with gene responses observed in this study suggest that environmental stress leads to increased protein damage, an adjustment of the proteome, and *de novo *synthesis of proteins in this species. Protein synthesis in ectotherms accounts for about 20 percent of the cellular energy budget [30,31]. *De novo *protein synthesis and protein repair in response to environmental stress are thus likely to incur substantial energetic costs in *P. moluccensis *and may reduce the energy available for other organismal functions such as growth and reproduction. These results are concordant with studies of the yeast *Saccharomyces cerevisiae *that have shown that yeast responds to a range of environmental stressors with a stereotypical change in gene expression and the suppression of around ten percent of assayed genes, many of which function in protein synthesis and cell growth [32].

A third group of gene functions commonly associated with environmental stress responses in *P. moluccensi*s included metabolic gene functions, in particular gene functions related to carbohydrate and lipid metabolism. Stress-responsive genes with metabolic functions included *SC4MOL*, *CHST2*, *amy2a*, *ACAD*, *dgat2l*, *dcxr*, and *fuca1*. Metabolic adjustments and the re-allocation of energy resources may reflect mechanisms that allow for increased levels of protein and cellular repair during stress.

Genes with stress-related functions, and functions in cellular repair, were also commonly associated with transcriptional stress responses in *P. moluccensis*, and included *dnajb11*, *peroxiredoxin 6*, *ptges3*, *apoea*, *hif1an*, and a gene encoding elastase 2. Elastase 2 hydrolyses collagen-IV and elastin and its activity has been shown to increase in the presence of reactive oxygen species [33]. Regulation of *elastase 2*, therefore, suggests increased levels of oxidative stress under conditions of environmental stress. In addition, genes with immune functions and the gene ontology 'response to pest, pathogen or parasite' were commonly associated with environmental stress responses, suggesting a challenge to the immune system. This functional category included the genes *CHST2*, T-cell receptor alpha, and haptoglobin. Finally, the gene function 'DNA repair' was significantly associated with many of the stress responses measured here, suggesting increased levels of DNA damage under conditions of environmental stress. Such responses are consistent with the observation that different types of stress can lead to immunosuppression [34,35] and genotoxic effects [36].

Here we have shown that a multi-stressor approach, and the identification of gene functions most often associated with gene expression responses to stress, can enhance our understanding of the biological processes altered by environmental stress. Our results suggest that common effects of environmental stress in *P. moluccensis *include suppression of cell growth, increased protein damage and *de novo *synthesis of proteins, metabolic adjustments and a reallocation of energy resources possibly related to increased protein and cellular repair, induction of stress genes and cellular repair systems, and a challenge of immune functions. Different types of stressors have been previously associated with a 'minimal stress proteome', comprised in particular of proteins involved in DNA, protein and molecular repair and energy metabolism [37]. Our results therefore provide experimental support for the presence of a stress proteome, and common stress responses, also in an ectothermic vertebrate, the coral reef fish *Pomacentrus moluccensis*.

### Early gene responses to environmental stress

In addition to the common stress responses discussed above, there were also differences between the responses to individual stressors. For example, there was a remarkable difference in the number of genes that were regulated in response to heat shock at 34°C in summer and winter. Mean winter and summer temperatures differ by approximately 4°C at the study site. Seasonal acclimation can lead to extensive transcriptional adjustments in teleosts [38-41] and may thus account for some of the transcriptional differences observed here. We cannot exclude, however, the possibility that some of the changes in gene expression were due to the male fish employed in the microarray analyses being at different stages of the reproductive cycle at the time of sampling. The different experimental designs employed in the summer and winter analyses (see Methods) may have further confounded these results as biological samples had been pooled in the winter analyses, but not in the summer analyses. Further studies will be required to more precisely define the sources of these differences in stress responses between summer and winter.

Many responses to hypoxia compensate for reduced cellular oxygen levels by increasing capacities for oxygen delivery, and by enhancing oxygen-independent ATP production by means of glycolysis [42]. The hypoxia-inducible transcription factor *HIF-1 *plays an important role in coordinating transcriptional responses to hypoxia [42-47]. In the present study, *HIF-1a *showed only limited gene regulation (data not shown) which is consistent with *HIF *regulation occurring largely at the protein level, not at the mRNA expression level. However, hypoxia-inducible factor 1 alpha subunit inhibitor (*HIF1an*), which modulates HIF-1 transcriptional activity, was significantly down-regulated in response to prolonged heat exposure (Additional File 1). While further data are required to understand the role of *HIF-1a *and *HIF1an *in response to hypoxic and heat stress in *P. moluccensis*, association of the gene ontologies 'glycolysis' and 'gluconeogenesis' with the response to hypoxia is consistent with the need to increase oxygen-independent ATP production. Furthermore, amongst the most significant expression responses to hypoxia was induction of *amy2a*, encoding the amylase-3 protein that endohydrolyses 1,4-α-D-glucosidic linkages in polysaccharides such as starch and glycogen to make dextrin, which can then be reduced to α-D-glucose. During hypoxia, this protein appears to free stored sugars for use in glycolysis and would, therefore, facilitate oxygen-independent ATP production.

### Gene expression responses to prolonged heat

Given current models of climatic change and the prediction of rising sea surface temperatures, we were particularly interested in gene responses to prolonged heat, and how early gene responses may change following prolonged exposure to heat. We found that while the gene ontology 'protein folding' remained associated with the response to heat even after prolonged exposure 'ubiquitin-dependent protein catabolism' was not. These results suggest that initial adjustments of the proteome during stress involve increased degradation of (stress-damaged) proteins and increased levels of ubiquitin-dependent protein catabolism. Over time, rates of protein catabolism appear to return to baseline levels, while protein chaperone systems remain induced.

Prolonged heat exposure was further associated with the gene ontology 'response to oxidative stress' and induction of the genes encoding peroxiredoxin 6 and apolipoprotein Ea, two proteins with antioxidant function. Oxidative stress is caused by the formation of reactive oxygen species and has been associated with many forms of stress [6,7,20,48-54]. It is likely that oxidative stress, and the associated cellular and molecular damage, incur substantial energetic costs during prolonged heat exposure. The fact that the gene ontologies 'carbohydrate metabolism' and 'lipid metabolism' remain associated with gene responses even after prolonged exposure to heat may reflect long-term re-allocation of energy resources. Such energetic re-organisation may compensate for the energetic needs associated with protein and cellular repair arising from prolonged heat exposure and the associated oxidative stress. For example, *ACAD*, *dgat2l*, *dcxr*, *HADH2*, *crabp2*, *Glb1*, *elovl6l*, *fuca1*, and *AGPAT3 *all encode proteins that function in carbohydrate or lipid metabolism and were regulated in response to prolonged heat.

Stress commonly suppresses immune function and chronic stress is typically associated with an increased risk of developing pathologies [34,35]. The association of 'response to pest, pathogen or parasite' with gene responses to prolonged heat suggests a continuous challenge of the immune system, and potentially, an elevated risk of disease. The ontology 'hemoglobin complex' was also associated with the response to prolonged heat suggesting that prolonged heat exposure may compromise oxygen supply. During heat stress, oxygen demand can exceed oxygen supply, leading to hypoxia in the cells [2,55]. Genes encoding hemoglobin beta embryonic-1, hemoglobin alpha embryonic-3, and haptoglobin were induced in response to prolonged heat in this study, likely reflecting this need to enhance oxygen delivery systems during heat stress. Our data suggest that prolonged heat exposure in the coral reef fish *P. moluccensis *is associated with oxidative stress, proteomic and metabolic adjustments, an induction of oxygen delivery systems, and a challenge of immune functions.

## Conclusion

Understanding thermal and environmental stress responses in coral reef fishes is paramount as current models of climate change predict a significant rise in sea surface temperatures within the next decades [56]. The predicted rise in sea surface temperatures adds to other anthropogenic threats, which have already significantly affected the functionality of coral reef ecosystems [57-59], and may lead to increased levels of physiological stress and mortality in coral reef organisms. For example, elevated sea surface temperatures a few degrees Celsius above normal can lead to coral bleaching [60]. While we have recently gained considerable knowledge about the relationship between sea surface temperatures and the incidence of coral bleaching, we presently have only limited data to estimate the potential effects of elevated temperatures on the physiology of coral reef fishes and, in particular, how heat alters biological function in these fishes. Our results provide the first evidence that the transcriptome and biological function of coral reef fishes can be significantly altered in response to a three-degree Celsius increase in temperature. Such a temperature rise is well within the range of predicted temperature increases of current climate change models. It is possible that the responses to heat observed here are altered following longer-term exposure. Further research is needed, therefore, to investigate the longer-term effects of increased temperatures on gene regulation as well as on growth, reproduction, and immune function. Since the functions of many heat-responsive genes are still unknown, experimental characterisation of gene functions during stress would also enhance our understanding of the effects of elevated temperatures on coral reef fishes and other ectothermic vertebrates. Future work may also establish closer links between the genome and phenome under conditions of environmental stress by relating liver gene responses to those observed in other tissues and by estimating the severity of stress using other indicators, such as blood cortisol levels or the expression of heat shock proteins. In addition, future work may investigate the changes in transcriptional regulation over the full time course of the biological response, from a few minutes to several weeks and months of exposure. Irrespective of the complexity of environmental stress responses discussed above, this study has demonstrated that there are a set of gene functions commonly associated with environmental stress responses in a warm-adapted ectothermic vertebrate, that these common responses are effectuated by a largely independent set of genes, and that the multi-stressor approach applied here is useful for delineating common responses from those unique to different types of environmental stress. A more complete understanding of environmental stress responses in ectothermic vertebrates will depend on the successful integration of environmental genomic data with data obtained from cellular, physiological and organismal studies, and our ability to unravel the complex interactions that occur between different levels of biological organisation.

## Methods

### Stress experiments

Summer-acclimated adult *P. moluccensis *were collected around Lizard Island, northern Great Barrier Reef, Australia (14°40'S, 145°28'E) by divers on SCUBA using barrier and scoop nets and transferred to the Lizard Island Research Station. Fish were housed in groups of up to 20 individuals in aquaria supplied with flow-through seawater at ambient temperature (approximately 28°C). Sections of PVC pipes and dead branching coral were provided for shelter. After two days of acclimation to these conditions, animals were transferred to individual aquaria of 31°C for five days or remained at ambient temperature (28°C) for five days. We exposed additional *P. moluccensis *individuals to heat shock at 31°C or 34°C, cold shock at 22°C, hypoxia (23–36% air saturation), or hyposmotic conditions (20 ppt salinity) for three hours. Hypoxic conditions were created by applying a constant flow of nitrogen to the tanks. Hyposmotic conditions were created by mixing seawater with distilled water, reducing the salinity of the seawater from 36 ppt to 20 ppt. The maximum range of naturally occurring environmental conditions for this population of coral reef fish are temperatures between 24°C in winter and 30°C in summer, air saturation around 100%, but possibly as low as 20% deep in branching coral at night [61], and salinities between 34 ppt and 36 ppt. All treatments were associated with behavioural changes such as increased ventilation rates and gasping for air at the surface. All experiments performed in this study, therefore, appear to have induced a physiological challenge in these experimental subjects and the gene responses observed are very likely to constitute a stress response.

It is practically impossible to measure transcriptional responses to a variety of stressors across the full time course of a biological response. Therefore, we have focused here on two time points of particular interest. The five-day heat exposure experiments targeted responses beyond the primary responses measured using only three-hour exposure. On any one day, an equal number of fish were subjected to either one of the stress treatments or transferred to aquaria at ambient conditions, in order to provide time-matched controls. We have previously performed a severe heat shock treatment at 34°C with winter-acclimated *P. moluccensis *[23]. We were thus able to compare summer and winter responses to severe heat shock (ambient conditions were 26°C in winter and 28°C in summer). Following these treatments, fish were killed by placing them on ice. Livers were excised immediately following killing of fish in order to avoid any potential decay of RNA and the RNA was stored in RNAlater™ (Qiagen) for microarray analysis. While we used liver for the analysis of gene expression responses, other tissues may have been affected differently. However, liver is a metabolically important tissue and should thus provide insight into a large number of transcriptional responses. We further assumed that gene expression changes measured using this approach are indicative of the types of biological functions affected by environmental stress, independent of how changes at the mRNA level may relate to changes in protein expression. Since age and gender can affect expression responses, we restricted the analyses of gene responses to males of similar standard length, assuming that similar standard length would indicate similar age. Hence, only adult male fish of standard length 47 ± 4 mm were used in the microarray analyses.

### Microarray analysis

The Compugen 16 K *D. rerio *oligonucleotide array was previously shown to be useful for studying gene responses in *P. moluccensis*. Quantitative real-time PCR confirmed differential gene expression for identified candidate genes and comparative genomic hybridisation experiments showed good cross-hybridisation for most genes between the two fish species [23]. This microarray represents one of the largest teleost microarrays available containing 16,399 oligos (65-oligomers) representing 15,806 unique *D. rerio *gene clusters plus controls. This array offered, therefore, the greatest chance of detecting large numbers of genes involved in transcriptional responses to stress. The arrays were printed by the Adelaide Microarray Facility. The list of genes immobilised on the array is available at [62].

Total RNA from liver tissue of stressed *P. moluccensis *and *P. moluccensis *kept at ambient conditions was extracted using TRIzol^® ^(Invitrogen) according to the manufacturer's instructions and purified using RNeasy™ columns (Qiagen), ethanol-precipitated and subsequently resuspended in nuclease-free water. The concentration and purity of RNA was determined by spectrophotometer readings at 260 and 280 nm. The integrity of the RNA was confirmed by agarose gel electrophoresis. All samples used in the microarray analyses were of highest RNA quality with no apparent signs of RNA degradation. Forty μg of total RNA were reverse transcribed and labelled using the SuperScript Plus Indirect cDNA Labeling System (Invitrogen) according to the manufacturer's instructions. Both oligo dT and random hexamers were used for priming of the reverse transcription reaction and Alexa Fluor^® ^555 and Alexa Fluor^® ^647 dyes (Invitrogen) were used for labelling. Prior to hybridisation, each microarray slide was immersed in distilled water at 60°C for 5 min and dried by centrifugation at 650 × g for 5 min. The purified fluorescently labelled cDNA samples were mixed with 5 μg human Cot-1 and 8 μg poly A, dried under reduced pressure, resuspended in 14 μl formamide and 14 μl of 6.25 × SSC, denatured by heating to 100°C for 3 min and transferred directly to ice. Finally, 0.6 μl of 10%(w/v) SDS was added to each sample. The probes were applied to the arrays and incubated at 42°C overnight in a humidified chamber. The arrays were washed in 0.5 × SSC containing 0.01%(w/v) SDS for 1 min, 0.5 × SSC for 3 min and 0.2 × SSC for 3 min. The slides were scanned using an Axon 4000B microarray scanner. Single image .tif files were saved for data analysis.

**Figure 4:**
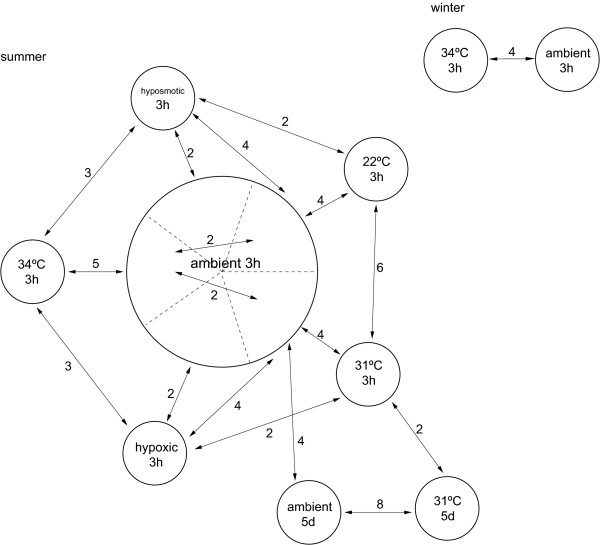
**Experimental design of the multi-stressor approach to investigate environmental stress responses**. The experiments were performed in an ectothermic vertebrate, the coral reef fish *Pomacentrus moluccensis*. Text within each circle names the stress and the duration it was applied, being three hours (3 h) or five days (5 d). Numbers above arrows indicate the number of microarray hybridisations used in the analysis of gene expression changes. Each microarray hybridisation compared relative expression levels in two samples (two-colour microarray analysis). Each sample in the microarray hybridisations represented one individual fish (no sample pooling) and a total of 118 individual fish were analysed in this study. Arrowheads indicate use of Alexa Fluor^® ^555 dye (green), which was swapped between samples to account for potential dye bias. The dotted lines within the ambient 3 h group indicate five groups of fish exposed to ambient conditions on separate days in order to provide time-matched controls for each of the stress treatments. Ambient conditions were 28°C in summer and 26°C in winter.

In total, 59 microarray hybridisations representing 118 individual *P. moluccensis *were performed using dye swaps and an approximately balanced design (Figure 4). While the aim of the study was to identify gene regulation in response to environmental stress using expression levels of fish kept at ambient conditions as reference, direct comparisons involving two stress treatments were included to make the experimental design more robust to the potential failure of some microarray hybridisations and to facilitate direct comparison between stress treatments. Based on the results of comparative genomic hybridisations, 985 of the 16,897 array spots were excluded from analysis of the microarray data because these spots showed poor cross-hybridisation between *P. moluccensis *and the *D. rerio *microarray [23]. For the remainder of the spots on the array, the red and green fluorescent signal intensities were extracted using SPOT software (CSIRO Mathematical and Information Sciences, Australia). The data were background corrected using the Spot morphological close/open method. Spot weights were calculated on the basis of the number of pixels in the spot, with spot areas between 30 and 300 pixels given full weight. The ratio of the resultant signal intensities (red/green) was log_2_-transformed. Statistical analyses were performed using the software package LIMMA [63] implemented in the R statistical software environment, following Smyth [64]. The transformed signal intensities of each grid on the array and global signal intensity were print-tip Loess normalised [65], and scale normalised between arrays. Loess normalisation subtracts a Loess regression curve from the MA-transformed data in order to remove dye bias from the data, while scale normalisation between arrays ensures that signal intensities are comparable across arrays. A moderated t-statistic was calculated for each gene on the array using an empirical Bayes method [64]. Benjamini and Hochberg's [66] method for controlling the false discovery rate (FDR) was used to control experiment-wide Type I error rates in the face of multiple testing. Genes with FDR-adjusted p-values < 0.1 were categorised as differentially expressed.

Within-ambient comparisons were performed to test whether fish treated on different days showed significantly different expression patterns (Figure 4). Pooling of ambient controls is justified where expression levels are consistent across them. Therefore, a linear model was fitted to the expression data with the ambient controls from the 31°C three hours treatment group used as a reference. Moderated t-statistics and F-statistics measuring the overall significance of the between-control contrasts were calculated [64]. Holm's method was used to adjust p-values for multiple testing [67]. The purpose here was to test whether there were significant differences between fish kept at ambient conditions on different days. Four genes had an adjusted p-value of less than 0.1. Three of these genes occupied neighbouring spots on the arrays. Two microarrays were outliers for these spots indicated by the residual M-value from the model. After removing these two arrays and refitting the linear model, only one spot remained significantly different amongst control groups. We decided to retain this microarray, but treat conclusions about this gene with caution. For the remaining analyses, ambient control samples were pooled and used as an ambient reference.

Gene annotation was performed using the program Resourcerer 12.0 [68]. Gene function of identified candidate genes was estimated by gene functions determined for *D. rerio *using a combination of AmiGo [69], iHop [70], and databases at NCBI [71].

### Identification of gene functions responsive to stress

To test whether genes of a certain function were more likely to be differentially expressed in response to different environmental stressors than expected if differential expression was independent of gene function, we used gene class testing and the gene set resampling (GSR) algorithm implemented in the software ermineJ [72]. In contrast to other commonly used gene class testing algorithms, the gene set resampling algorithm does not require a threshold for gene selection. Instead, all genes belonging to a particular gene ontology class are used to compute a raw score r = -∑i log(pi), where pi is the p-value for differential expression for each gene in the gene ontology class. Thus, GSR employs the continuous evidence contained in the p-values for differential expression and is the method of choice for gene class testing where there is no *a priori *gene grouping information. Gene ontology classes of size k = 5–100 were examined. For genes that were represented multiple times on the array, the minimum p-value was used. In order to calculate the distribution of raw scores under the null hypothesis of random distribution of gene ontologies, a random set of genes of the same size as each of the gene classes of interest was drawn from the data and the raw score r was computed for the random set. We performed one million iterations of this procedure. The significance for a gene set class was calculated as the fraction of random trials resulting in a score higher than r and the resulting p-values for overall significance were FDR-corrected.

### Hierarchical clustering and visualisation of gene responses across treatments

Unsupervised hierarchical clustering and the software programs Cluster and TreeView [73] were used to organise differentially expressed genes into groups of co-regulated genes. Array- and gene-normalised values of the t-statistic and complete linkage uncentered correlation were employed for clustering. The values of the t-statistic were chosen over average M-values, because the t-statistic not only reflects the magnitude of the average expression change, but also retains information on the variability in expression response across biological replicates. Visualisation of M-values representing individual arrays was not appropriate in this study because both direct and indirect comparisons were used for estimation of stress expression responses and indirect comparisons could not be unambiguously assigned to individual stress treatments.

To test for a relationship between cluster identity and gene function, we performed over-representation analysis (ORA) implemented in the software ermineJ [72]. The ORA algorithm is most appropriate here because the genes for this analysis naturally fell into two groups, being either in the cluster of interest or not. Gene functions of genes that were part of the cluster in question were compared to the gene functions of all genes represented on the microarray and we tested for a significant over-representation of gene functions amongst genes within a cluster.

## Competing interests

The author(s) declares that there are no competing interests.

## Authors' contributions

KSK, RHC and MJC conceived and designed this study. KSK and GS performed the statistical analyses. ACW provided the microarrays for use in this study. KSK performed the field and microarray experiments and prepared the manuscript. RHC, MJC, GS, and ACW helped to draft the manuscript. All authors read and approved the final manuscript.

## Supplementary Material

Additional file 1 **Changes in mRNA levels in *Pomacentrus moluccensis *exposed to three-hour stress treatments compared to *P. moluccensis *kept at ambient conditions**. GenBank accession numbers refer to the *Danio rerio *clones represented on the microarray. Statistical significance was determined using Bayesian analysis of the expression response across biological replicates (p-values are FDR-corrected). Negative values of fold change indicate down-regulation of gene in stressed *P. moluccensis*, while positive values indicate up-regulation. Only genes with FDR-corrected p-values < 0.1 and genes with greater than two-fold expression changes are reported here. Ambient conditions were 28°C, 100% air saturation, and 36 ppt salinity. Click here for file

Additional file 2 **Changes in mRNA expression levels in heat-stressed *Pomacentrus moluccensis*, exposed to elevated temperatures (31°C) for five days compared to *P. moluccensis *kept at ambient temperature (28°C) for five days**. Only genes for which information regarding gene function is currently available are reported here. Where multiple functions have been identified for a gene, the gene function most relevant in the context of this study is reported. GenBank accession numbers refer to the *Danio rerio *clones represented on the microarray. Genes were ranked according to statistical significance as determined by Bayesian analysis of the expression response across biological replicates. Negative values of fold change indicate down-regulation of a gene in heat-stressed *P. moluccensis*, while positive values indicate up-regulation (p-values are FDR-corrected). Click here for file
