# Supplementary material for: From transcriptome to biological function: environmental stress in an ectothermic vertebrate, the coral reef fish Pomacentrus moluccensis
Source: BMC Genomics. 2007 Oct 5;8:358. doi: 10.1186/1471-2164-8-358 (PMC2222645; doi:10.1186/1471-2164-8-358)
Supplement: Additional file 1 — Changes in mRNA levels in Pomacentrus moluccensis exposed to three-hour stress treatments compared to P. moluccensis kept at ambient conditions. GenBank accession numbers refer to the Danio rerio clones represented on the microarray. Statistical significance was determined using Bayesian analysis of the expression response across biological replicates (p-values are FDR-corrected). Negative values of fold change indicate down-regulation of gene in stressed P. moluccensis, while positive values indicate up-regulation. Only genes with FDR-corrected p-values < 0.1 and genes with greater than two-fold expression changes are reported here. Ambient conditions were 28°C, 100% air saturation, and 36 ppt salinity. [file 1471-2164-8-358-S1.pdf]

Additional File 1 Changes in mRNA levels in *Pomacentrus moluccensis* exposed to three-hour stress treatments compared to *P. moluccensis* kept at ambient conditions (28ºC, 100% air saturation). GenBank accession numbers refer to the *Danio rerio* clones represented on the microarray. Statistical significance was determined using Bayesian analysis of the expression response across biological replicates (p-values are FDR-corrected). Negative values of fold change indicate down-regulation of gene in stressed *P. moluccensis*, while positive values indicate up-regulation. Only genes with FDR-corrected p-values <0.1 and genes with greater two-fold expression changes are reported here.

Rank	GenBank Accession *D.rerio* clone	UniGene ID	Gene Symbol	Putative Identification	Function	Fold Change	p
**Cold shock (22ºC)**							
1	BI533884	Dr.17570	mknk2	MAP kinase-interacting kinase 2	Response to stress; Protein kinase cascade	+2.20	0.1667
16	AI601458	Dr.17520	ube2h	Ubiquitin-conjugating enzyme E2H	Protein catabolism; Response to heat	+2.13	0.9994
**Moderate heat shock (31ºC)**							
1	AW134054	Dr.36134	plec1	Plectin 1	Structural constituent of cytoskeleton	+1.75	0.0015
9	AI601458	Dr.17520	ube2h	ubiquitin-conjugating enzyme E2H	Protein catabolism; Response to heat	+2.44	0.2658
72	BI710730	Dr.31497	ncl	Nucleolin	RNA binding	+2.78	0.5414
170	AW116649	Dr.1099	prkcsh	Protein kinase C substrate 80K-H	Kinase activity; Calcium ion binding	-2.05	0.5604
172	AI476925	Dr.1218	EXOSC2	Exosome component 2	Exonuclease activity; rRNA processing	+2.15	0.5604
186	AB026980	Dr.8092	odz4	odd Oz/ten-m homolog 4	MAPKKK cascade; immune response	+2.30	0.5604
315	BI673605	Dr.6875	TRIAD3	TRIAD3 protein	Protein binding; Ubiquitin cycle	+2.57	0.6326
353	BI865754	Dr.59783	hmg4	High mobility group protein 4	DNA-dependent regulation of transcription	+2.28	0.6608
464	AW826769	Dr.32001	ptk9l	Protein tyrosine kinase 9-like	Cytoskeleton; Actin binding	+2.05	0.7174
1039	AB057355	Dr.12576	ednra	Endothelin receptor type A	Signal Transduction	+2.14	0.7895
**Severe heat shock (34ºC)**							
1	AF387900	Dr.6509	pdip5	Protein disulfide isomerase-related protein P5	Protein folding	+4.89	0.0000
2	AI959074	Dr.18762	rhoA	Ras-like protein RhoA	Regulation of cell cycle	-1.60	0.0006
3	BG729495	Dr.31651	ZCCHC11	Zinc finger CCHC domain containing 11 isoform b	DNA replication, recombination, and repair	+1.99	0.0039
4	AW175080	Dr.8390	SC4MOL	Sterol-C4-methyl oxidase-like	Catalytic activity; Sterol biosynthesis	-1.66	0.0624
5	AW279775	Dr.3130	rgs4	Regulator of G-protein signalling 4	Signal transducer activity	-1.30	0.0624
6	BI876768	Dr.43918	znf395	Zinc finger protein 395	DNA-dependent regulation of transcription	-1.52	0.0624
197	AF246169	Dr.30471	tcra	T-cell receptor alpha variable region	T-cell receptor complex	-2.25	0.9998
845	BE557072	Dr.10066	birc6	Baculoviral IAP repeat-containing 6	Ubiquitin conjugating enzyme activity	-2.49	0.9998
**Hypoxia (23-36% air saturation)**							
1	AI558655	Dr.36283	acin1a	Apoptotic chromatin condensation inducer 1a	DNA binding; Apoptosis	+2.47	0.0008
2	AI522803	Dr.34133	Ela2	Elastase 2	Proteolysis and peptidolysis	+2.90	0.0011
3	AI558632	Dr.33926	ElaA	Elastase A	Proteolysis and peptidolysis	+2.99	0.0085
5	BI476356	Dr.16450	CHST2	Carbohydrate sulfotransferase 2	Inflammatory response; Sulfur metabolism	+1.32	0.0085
7	BM183382	Dr.16654	AVD	Avidin	Biotin binding	+2.25	0.0176
8	AI496860	Dr.31546	Cpa2	Carboxypeptidase A2	Proteolysis and peptidolysis	+2.84	0.0258
9	AF025305	Dr.1109	bactin2	Actin, cytoplasmic 1 (Beta-actin)	Structural constituent of cytoskeleton	+1.60	0.0647
10	BM103972	Dr.2437	amy2a	Amylase-3 protein	Carbohydrate metabolism; Hydrolase activity	+2.76	0.0651
16	AW777717	Dr.2317	NKX2-8	NK2 transcription factor related, locus 8	Transcription factor activity	-2.19	0.2928
112	AF052252	Dr.21004	fdk9	Fork head domain protein FKD9	Transcription factor activity	-2.38	0.6438
332	AB026980	Dr.8092	odz4	Odd Oz/ten-m homolog 4	MAPKKK cascade; immune response	-2.06	0.9476
578	BI710730	Dr.31497	ncl	Nucleolin	RNA binding	-2.01	0.9998
