# Supplementary material for: From transcriptome to biological function: environmental stress in an ectothermic vertebrate, the coral reef fish Pomacentrus moluccensis
Source: BMC Genomics. 2007 Oct 5;8:358. doi: 10.1186/1471-2164-8-358 (PMC2222645; doi:10.1186/1471-2164-8-358)
Supplement: Additional file 2 — Changes in mRNA expression levels in heat-stressed Pomacentrus moluccensis, exposed to elevated temperatures (31°C) for five days compared to P. moluccensis kept at ambient temperature (28°C) for five days. Only genes for which information regarding gene function is currently available are reported here. Where multiple functions have been identified for a gene, the gene function most relevant in the context of this study is reported. GenBank accession numbers refer to the Danio rerio clones represented on the microarray. Genes were ranked according to statistical significance as determined by Bayesian analysis of the expression response across biological replicates. Negative values of fold change indicate down-regulation of a gene in heat-stressed P. moluccensis, while positive values indicate up-regulation (p-values are FDR-corrected). [file 1471-2164-8-358-S2.pdf]

Additional File 2 Changes in mRNA expression levels in heat-stressed *Pomacentrus moluccensis*, exposed to elevated temperatures (31ºC) for five days compared to *P. moluccensis* kept at ambient temperature (28ºC) for five days. Only genes for which information regarding gene function is currently available are reported here. Where multiple functions have been identified for a gene, the gene function most relevant in the context of this study is reported. GenBank accession numbers refer to the *Danio rerio* clones represented on the microarray. Genes were ranked according to statistical significance as determined by Bayesian analysis of the expression response across biological replicates. Negative values of fold change indicate down-regulation of gene in heat-stressed *P. moluccensis*, while positive values indicate up-regulation (p-values are FDR-corrected).

Rank	GenBank Accession *D.rerio* clone	UniGene ID	Gene Symbol	Putative Identification	Function	Fold Change	p
Cell Adhesion							
21	AF081128	Dr.28760	fn1	Fibronectin 1	Protein binding; Cell adhesion	+2.09	0.0227
45	AI641045	Dr.9212	DKEYP-18C4.2	Podocalyxin precursor	Negative regulation of cell adhesion	+1.93	0.0386
72	AF081128	Dr.28760	fn1	Fibronectin 1	Protein binding; Cell adhesion	+1.90	0.0414
81	BI882735		nrxn3	Neurexin 3-alpha	Cell adhesion	+1.28	0.0428
161	BI846718		papln	Papilin, proteoglycan-like sulfated glycoprotein	Cell adhesion; Protein binding	+1.61	0.0614
203	BI891338			Protocadherin 1 isoform 1 precursor	Cell adhesion; Calcium ion binding	+1.56	0.0737
211	AW019629	Dr.6507	eva1	Epithelial V-like antigen 1	Cell adhesion; Protein binding	+1.35	0.0752
255	AI384393	Dr.418	pcdh2g9	Protocadherin 2 gamma 9	Homophilic cell adhesion	-1.26	0.0907
Cell Cycle and Cell Growth							
26	BG308696	Dr.11045	GRIM19	Cell death-regulatory protein GRIM19	Apoptosis	+1.75	0.0227
33	BG308520	Dr.33755	TP53INP1	Tumor protein p53 inducible nuclear protein 1	Apoptosis	+1.54	0.0300
61	BI672829	Dr.13625	slah21	Seven in absentia homolog 2 (Drosophila)-like	Mitotic checkpoint	+1.61	0.0392
70	AF229449	Dr.8287	jag2	Jagged 2 isoform 1	Cell cycle; Cell communication	+1.36	0.0392
100	BI472637	Dr.15050	SEPT3	Septin 3	Cell cycle; Cytokinesis	+1.35	0.0504
144	AI959372	Dr.1212	ccng1	Cyclin G1	Cell cycle checkpoint	+1.34	0.0614
173	AW154091	Dr.33730	cdca3	Cell division cycle associated 3 isoform 1	Cell division	+1.35	0.0667
205	BI672089	Dr.14498	TXNL4B	Thioredoxin-like 4B	Mitosis	+1.99	0.0745
285	BG727086	Dr.41921	TGFB1	Transforming growth factor beta-1-binding protein	Cell Growth; Inflammatory response	+1.33	0.0960
288	BI672476	Dr.14472		Amyloid-like protein 2 precursor	Apoptosis; Cell adhesion; Heparin binding	+1.29	0.0965
307	BI672424	Dr.14459	bax	BCL2-associated X protein	Regulation of apoptosis; Cell cycle	+1.38	0.0975
Cytoskeleton							
46	BM172691	Dr.14192	DNAH11	Dynein, axonemal, heavy polypeptide 11	Microtubule motor activity	+1.53	0.0386
57	BM025904	Dr.6441	DNAH11	Dynein, axonemal, heavy polypeptide 11	Microtubule motor activity	+1.22	0.0392
126	AW116733	Dr.19450	SPTB	Spectrin	Structural constituent of cytoskeleton	+1.63	0.0586
147	BI979131	Dr.13282	DNAL4	Dynein, axonemal, light polypeptide 4	Microtubule-based movement	+1.54	0.0614
148	AI584969	Dr.28264	gas8	Growth arrest-specific 8	Cytoskeleton	+1.31	0.0614
197	BI864873	Dr.6173	tubb2	Tubulin beta-2	Structural constituent of cytoskeleton	+1.50	0.0710
231	BI889708	Dr.1964	PTPN13	Protein tyrosine phosphatase, non-receptor type 13	Cytoskeleton; Protein binding	+1.28	0.0804
239	AA658732	Dr.867	wdr1	WD repeat domain 1	Cytoskeleton	+1.36	0.0830
244	BI671622		filamin 2	Gamma Filamin	Cytoskeleton; Actin binding	+1.28	0.0887

Table 1 cont.

Rank	GenBank Accession *D.rerio* clone	UniGene ID	Gene Symbol	Putative Identification	Function	Fold Change	p
Metabolism							
35	BF156211	Dr.11446	ACAD	Acyl-CoA dehydrogenase	Electron transport; Metabolism	-1.25	0.0311
48	BI892323	Dr.30594	dgat2l	Diacylglycerol acyltransferase 2-like	Lipid metabolism	+1.41	0.0386
59	AA494790	Dr.396	dcxr	Dicarbonyl/L-xylulose reductase	Glucose metabolism	+1.39	0.0392
65	AW305632	Dr.36924	HADH2	3-hydroxyacyl-CoA dehydrogenase type II	Oxidoreductase activity; Lipid metabolism	+1.54	0.0392
78	BI710519	Dr.29442	crabp2	Cellular retinoic acid binding protein 2	Lipid binding	+1.22	0.0418
103	BM104068		wac	WW domain containing adaptor with coiled-coil	Glucan 1,4-alpha-glucosidase activity	+1.26	0.0527
107	AI545065	Dr.7466	ak3l1	Adenylate kinase 3-like 1	ATP binding; Adenylate kinase activity	+1.52	0.0558
118	AW281616	Dr.12080	Glb1	Beta-galactosidase	Carbohydrate metabolism	+1.30	0.0586
127	AW116000	Dr.7640	setd8	SET domain containing (lysine methyltransferase) 8	Methyltransferase activity	+1.44	0.0586
186	AI957831	Dr.15140	mettl6	Methyltransferase-like 6	Methyltransferase activity	+1.91	0.0698
188	BM103899	Dr.12654	elovl6l	ELOVL family member 6	Fatty acid elongation	+1.33	0.0698
219	BM024412	Dr.2359	nvl	Nuclear VCP-like	ATP binding	+1.53	0.0792
221	BI885898		dnmt	DNA (cytosine-5-)-methyltransferase 3	Methyltransferase activity; DNA methylation	+1.33	0.0792
234	AW280062	Dr.9165	ZDHH9	Zinc finger, DHHC-type containing 9	Metal ion binding; Transferase activity	-1.23	0.0821
250	BI882221	Dr.33925	rdh12l	Odd Oz/ten-m homolog 3; retinol dehydrogenase 12, like	Oxidoreductase activity	+1.46	0.0900
278	AW115782	Dr.6619	pgd	Phosphogluconate hydrogenase	Oxidoreductase; Pentose-phosphate shunt	-1.26	0.0959
281	AW232289	Dr.1041	fuca1	Fucosidase, alpha-L- 1, tissue	Carbohydrate metabolism	+1.47	0.0959
286	BM070575	Dr.11133	dpysl5b	Dihydropyrimidinase-like 5b	Hydrolase activity	+1.26	0.0960
300	AW232323	Dr.30175	AGPAT3	1-acylglycerol-3-phosphate O-acyltransferase 3	Acyltransferase activity	+1.48	0.0968
Protein Processing							
2	AW116649	Dr.1099	prkcsh	Protein kinase C substrate 80K-H	Protein kinase cascade	+3.87	0.0131
18	BM102551	Dr.17149	PAPPA	Pregnancy-associated plasma protein A	Proteolysis	+2.06	0.0195
36	BI672058	Dr.2642	ntf2	Nuclear transport factor 2	Protein transport	+1.68	0.0317
74	AF130460	Dr.8275	trpc4apb	Transient receptor potential cation channel	Protein transport	+1.49	0.0414
86	BG306270	Dr.15263	snrk	SNF1-related kinase	Protein kinase activity	+1.57	0.0441
91	AW170941	Dr.7993	USP29	Ubiquitin specific peptidase 29	Ubiquitin-dependent protein catabolism	-1.25	0.0464
102	BI704448	Dr.17040	chm	Rab escort protein 1; choroideremia	Intracellular protein transport	+2.31	0.0509
108	BM101665	Dr.9559	ctss	Cathepsin S	Proteolysis	-1.39	0.0563
113	BI886677	Dr.18008	RBM12	RNA binding motif protein 12	Protein binding	-1.17	0.0583
130	BI979883	Dr.31059	psmc3	Proteasome (prosome, macropain) 26S subunit, ATPase, 3	Ubiquitin-dependent protein catabolism	-1.34	0.0586
132	BI891871	Dr.3615	csnk1d	Casein kinase 1, delta	Protein serine/threonine kinase activity	+1.34	0.0587
140	AW134164	Dr.20940	fbxl12	F-box and leucine-rich repeat protein 12	Ubiquitin-dependent protein catabolism	+1.63	0.0587
145	AW019421	Dr.6291	tnpo2	Transportin 2 (importin 3, karyopherin beta 2b)	Protein transport	-1.27	0.0614
164	AI883718		ATG7	ATG7 autophagy related 7 homolog	Positive regulation of protein modification	+1.85	0.0625
175	AI588515	Dr.2974	Rabac1	Rab acceptor 1 (prenylated)	Protein binding; Golgi apparatus	+1.34	0.0667
178	BG737266		ru2	Ruby eye2-like protein	Protein binding	+1.35	0.0667
200	BG728947	Dr.12053	COG2	Component of oligomeric golgi complex 2	Protein transport	-1.18	0.0732
218	BG737844		mark1	MAP/microtubule affinity-regulating kinase 3	Protein kinase activity	+1.37	0.0789
259	BG302807	Dr.28615	snx12	Sorting nexin 12	Protein transport	-1.26	0.0914

Table 1 cont.

Rank	GenBank Accession *D.rerio* clone	UniGene ID	Gene Symbol	Putative Identification	Function	Fold Change	p
Protein Processing cont.							
267	BG304171	Dr.20155	ss18	Synovial sarcoma translocation, chromosome 18	Protein binding	+1.55	0.0936
279	AW058757	Dr.20362	cdc7	Cell division cycle 7-related protein kinase	Protein serine/threonine kinase activity	-1.17	0.0959
290	BI704278	Dr.26555	ctsla	Cathepsin L, a	Proteolysis and peptidolysis	+1.35	0.0965
301	BI980628	Dr.16147	osgepl1	Novel glycoprotease; O-sialoglycoprotein endopeptidase-like 1	Proteolysis and peptidolysis	+1.31	0.0968
323	BI882056	Dr.6513	cyhr1	Cysteine and histidine rich 1	Protein binding	+1.30	0.0998
Response to stress							
3	AF082662	Dr.28283	hbbe1	Hemoglobin beta embryonic-1	Oxygen transport	+1.86	0.0131
54	AW344134	Dr.9667	dnajb11	DnaJ (Hsp40) homolog, subfamily B, member 11	Heat shock protein binding; Protein folding	+1.82	0.0392
60	BI673277	Dr.2704	stambp	Associated molecule with the SH3 domain of STAM	Anti-apoptosis; Ubiquitin cycle	+1.27	0.0392
67	AA605696	Dr.13845	prdx6	Peroxiredoxin 6	Response to reactive oxygen species	+1.53	0.0392
75	AI884178	Dr.4306	clpx	Caseinolytic peptidase X homolog (E. coli)	Protein folding	+1.63	0.0414
128	AF082662	Dr.28283	hbbe1	Hemoglobin beta embryonic-1	Oxygen transport	+1.65	0.0586
224	AI964223	Dr.2970	apoea	Apolipoprotein Ea	Induction of apoptosis; Antioxidant activity	+1.31	0.0792
230	BM185394	Dr.14011	PFDN4	Prefoldin subunit 4	Protein folding	+1.29	0.0804
235	AI793830	Dr.31082	ptges3	Prostaglandin E synthase 3 (cytosolic)	Hsp90 binding; Protein folding	+1.34	0.0821
256	AI353083	Dr.1450	hbae3	Hemoglobin alpha embryonic-3	Oxygen transport	+1.90	0.0914
282	AF246176	Dr.30472		T-cell receptor alpha variable region	Immune Response; Receptor Activity	+1.37	0.0959
296	BG884044	Dr.4867	HP	Haptoglobin	Defense response; Hemoglobin binding	+1.27	0.0965
298	BI890693	Dr.13371	hif1an	Hypoxia-inducible factor 1, alpha subunit inhibitor	Oxidoreductase; Regulation of transcription	-1.23	0.0966
Signal Transduction							
17	BM183950	Dr.14422	Cdon	Cell adhesion molecule-related/down-regulated by oncogenes	Smoothened signaling pathway	+1.46	0.0195
40	BM184012	Dr.3319	CALM3	Calmodulin 3 (phosphorylase kinase, delta)	G-protein coupled receptor protein signaling	+1.23	0.0368
43	BG306148	Dr.16542	GRWD1	Glutamate-rich WD repeat containing 1	Signal transduction	+1.85	0.0386
55	AF083382	Dr.8112	sema3ab	Semaphorin 3ab	Cell-cell signaling	+2.14	0.0392
66	AF116853	Dr.8085	frzb	Frizzled-related protein	Wnt receptor signaling pathway	+1.54	0.0392
104	BF938356	Dr.11228	arl2bp	ADP-ribosylation factor-like protein 2	Small GTPase regulator activity	+1.58	0.0527
109	AW171604	Dr.8015	Cnksr2	Connector enhancer of kinase suppressor of Ras 2	Regulation of signal transduction	-1.24	0.0583
129	AW128372	Dr.7282	IQGAP2	IQ motif containing GTPase activating protein 2	Small GTPase mediated signal transduction	+1.54	0.0586
137	AW115682	Dr.7257	rasgef1b	RasGEF domain family, member 1B	Small GTPase mediated signal transduction	+1.29	0.0587
142	AW115765	Dr.9538	NCK2	NCK adaptor protein 2	Signal complex formation	-1.26	0.0609
163	AJ007742	Dr.8054	ptc2	Patched2	Hedgehog receptor activity	+1.36	0.0625
168	BG307536	Dr.9665	rhoG	Ras homolog gene family, member G	Signal Transduction	-1.33	0.0649
176	BI886464	Dr.25497	szl	Sizzled	BMP signaling pathway	+1.69	0.0667
181	BM183274	Dr.15125		Small inducible cytokine subfamily A	Signal Transduction	+1.27	0.0667
185	U49405		FZD4	Frizzled homolog 4	Frizzled signaling pathway	+1.29	0.0696
207	AW170891	Dr.35466	IGBP1	Immunoglobulin (CD79A) binding protein 1	Signal transduction	+1.43	0.0752
222	BI878477	Dr.10893	sara2	SAR1a gene homolog 2 (S. cerevisiae)	Small GTPase mediated signal transduction;	+1.31	0.0792
229	AF105152	Dr.354	rho	Rhodopsin	Rhodopsin mediated signaling	+1.33	0.0800
246	AI477343	Dr.2419	tiaf1	TGFB1-induced anti-apoptotic factor 1	I-kappaB kinase/NF-kappaB cascade	-1.18	0.0899

Table 1 cont.

Rank	GenBank Accession *D.rerio* clone	UniGene ID	Gene Symbol	Putative Identification	Function	Fold Change	p
Signal Transduction cont.							
251	AW078288	Dr.32732	invs	Inversin	Wnt receptor signaling pathway	+1.24	0.0900
252	AI965042	Dr.4874	rab1a	RAB1A, member RAS oncogene family	Two-component signal transduction system	+1.55	0.0900
268	BM183955	Dr.2109	rap1a	Ras-related protein RAP-1A	Signal transduction	-1.27	0.0952
297	AW116767	Dr.4451	chp	Calcium binding protein p22	Calcium-mediated signaling; Transcytosis	-1.23	0.0966
303	BE557009	Dr.28754	PRKAR1A	cAMP-dependent protein kinase type I-alpha regulatory chain	Intracellular Signaling Cascade	+1.20	0.0969
317	AW116392	Dr.6091	rhoQ	Ras homolog gene family, member Q	Signal transduction	-1.19	0.0997
Transcription							
7	BM156785	Dr.15390	FOXL1	Forkhead box protein L1	DNA-dependent regulation of transcription	+1.81	0.0164
8	BI897147	Dr.29850	runx1	Runt-related transcription factor 1	DNA-dependent regulation of transcription	-1.79	0.0164
10	BM072263	Dr.16724	znf238	BTB/POZ domain protein; Zinc finger protein 238	DNA-dependent regulation of transcription	+1.72	0.0166
13	U24225	Dr.625	Snail2	Snail2	Transcription factor activity	-1.37	0.0170
14	AW077156	Dr.27746	RBMS3	RNA binding motif, single stranded interacting protein	RNA binding	+1.64	0.0170
20	BM026839	Dr.9702	rmb19	RNA binding motif protein 19	Nucleic acid binding	+1.34	0.0218
22	BM024211	Dr.6364	ppp1r10	Protein phosphatase 1, regulatory subunit 10	Nucleic acid binding; Defense response	+1.71	0.0227
29	BI887656	Dr.356	gata2	GATA-binding protein 2	Transcription factor activity; Heme binding	-1.30	0.0238
39	BM182314	Dr.14269	tmf1	TATA element modulatory factor 1	DNA-dependent regulation of transcription	+1.31	0.0365
44	X65060		dlx3	Distal-less homeobox 3	DNA-dependent regulation of transcription	+2.53	0.0386
52	U14592	Dr.334	otx2	Orthodenticle homolog 2 (homeobox protein OTX2)	DNA-dependent regulation of transcription	+2.00	0.0392
62	AJ344448	Dr.12575	dcp1a	Decapping enzyme	Positive regulation of transcription	+1.80	0.0392
64	BI889395		CNOT6L	CCR4-NOT transcription complex, subunit 6-like	RNA processing and modification	+1.57	0.0392
77	AW018998	Dr.4008	msl-1	Male-specific lethal-1 protein	Chromatin binding; Dosage compensation	-1.28	0.0418
85	BG303824	Dr.14895	ZNF135	Zinc finger protein 135	DNA-dependent regulation of transcription	-1.27	0.0441
98	U84616	Dr.2328	elf2	E74-like factor 2 (ets domain transcription factor)	DNA-dependent regulation of transcription	+1.66	0.0475
111	BI878117	Dr.85	nucb2a	Nucleobindin 2a	DNA binding; Calcium ion binding	+1.39	0.0583
116	BI430221	Dr.37036	SLU7	Step II splicing factor SLU7	mRNA splice site selection	+1.33	0.0586
133	AI601470	Dr.35889	hcfc1	Host cell factor C1 (VP16-accessory protein)	Regulation of transcription; Cell cycle	+1.23	0.0587
141	AW170975	Dr.36935	ilf3	Interleukin enhancer binding factor 3	DNA-dependent regulation of transcription	+1.41	0.0594
155	BG306387	Dr.11261	A2bp1	Ataxin 2-binding protein 1	RNA binding	+1.74	0.0614
156	BI891001	Dr.29941	top2a	DNA topoisomerase II	DNA unwinding during replication	+1.35	0.0614
157	BI866527	Dr.13962	gins3	GINS complex subunit 3	DNA-dependent DNA replication	-1.18	0.0614
158	AW305388	Dr.10033	lsm7	LSM7 homolog, U6 small nuclear RNA associated	Nuclear mRNA splicing, via spliceosome	+1.39	0.0614
160	BM096095	Dr.361	seph	Selenoprotein H	DNA binding	+1.41	0.0614
162	BI891601	Dr.17679	taf12	TAF12 RNA polymerase II,	DNA-dependent regulation of transcription	+1.27	0.0618
169	AW116245	Dr.14278	znf131	Zinc finger protein 131	DNA-dependent regulation of transcription	+1.32	0.0655
172	BM095242	Dr.14888	PRPF8	U5 snRNP-specific protein (220kDa)	Nuclear mRNA splicing, via spliceosome	-1.35	0.0667
179	BI877633	Dr.19658	Tip5	TTF-I interacting peptide 5	DNA-dependent regulation of transcription	+2.48	0.0667
184	BI984001	Dr.24310	zcchc17	Zinc finger, CCHC domain containing 17	Nucleic acid binding; Zinc ion binding	+1.31	0.0696
190	AF071268		hoxd9a	Homeobox protein D9a	DNA-dependent regulation of transcription	+1.36	0.0698
191	AJ293862		sall1a	Danio rerio partial sall1a gene for putative spalt protein.	Nucleic acid binding; Zinc ion binding	+1.25	0.0698
196	BI892074	Dr.9145	H3F3B	H3 histone, family 3B	DNA binding	+1.26	0.0710

Table 1 cont

Rank	GenBank Accession *D.rerio* clone	UniGene ID	Gene Symbol	Putative Identification	Function	Fold Change	p
Transcription cont.							
204	BI887415	Dr.4758		Proline-rich protein	Nucleic acid binding	+1.35	0.0745
208	BM156999	Dr.1992	lmx1b	LIM/homeobox protein LMX1B	Transcription factor activity	+1.29	0.0752
210	AI558282	Dr.21124	Sertad2	SERTA domain containing 2	DNA-dependent regulation of transcription	-1.23	0.0752
223	AW171228	Dr.115	tardbpl	TAR DNA binding protein, like	DNA-dependent regulation of transcription	-1.29	0.0792
237	BI890768		znf235	Zinc finger protein 93 homolog	DNA-dependent regulation of transcription	+1.34	0.0821
245	BI878611	Dr.8928	cnot8	CCR4-NOT transcription complex, subunit 8	DNA-dependent regulation of transcription	-1.31	0.0896
261	AI957820	Dr.27315	fen1	Flap structure-specific endonuclease 1	DNA repair	+1.31	0.0914
262	AF168008	Dr.618	pea3	ETS-domain transcription factor pea3	DNA-dependent regulation of transcription	+1.51	0.0914
266	AW420369	Dr.27962	scml2	Sex comb on midleg-like 2	DNA-dependent regulation of transcription	+1.47	0.0936
302	X65060		dlx3	Distal-less homeobox 3	DNA-dependent regulation of transcription	+1.26	0.0969
308	AF071240		hoxa11a	Homeobox protein A11a	DNA-dependent regulation of transcription	+1.24	0.0975
Translation							
4	BM181896	Dr.33915	rps6	40S ribosomal protein S6	Protein biosynthesis	+1.32	0.0131
32	BI889409	Dr.14812	mrpl36	Mitochondrial ribosomal protein L36	Structural constituent of ribosome	+1.43	0.0298
34	BM071666	Dr.16980	mrpl30	Mitochondrial ribosomal protein L30	Structural constituent of ribosome	+1.33	0.0302
97	AI667224	Dr.34694	EIF2C1	Eukaryotic translation initiation factor 2C 1	Protein biosynthesis	+1.60	0.0475
131	AI964245	Dr.7888	rrs1	Ribosome biogenesis regulatory protein	Ribosome biogenesis and assembly	+1.32	0.0587
276	AW232304	Dr.4626	eif2b2	Eukaryotic translation initiation factor 2B, subunit 2 beta	Protein biosynthesis	+1.43	0.0959
283	AI545168	Dr.48984	DDX3	DEAD-box protein 3	Nucleic acid binding	+1.26	0.0959
299	AW117050	Dr.2876	EIF4B	Eukaryotic translation initiation factor 4B	Protein biosynthesis	+1.35	0.0966
Transport							
79	AW076983	Dr.7965	clcn4	Chloride channel 4	Chloride transport	+1.39	0.0418
171	AI793405	Dr.4160		Collagen alpha 1	Phosphate transport; Skeletal development	+1.31	0.0667
233	AI667527	Dr.5169	abcb7	ATP-binding cassette, sub-family B, member 7	Transport; ATPase activity	+1.27	0.0815
Other							
30	BI350560	Dr.34580	CRTAC1	Cartilage acidic protein 1	Calcium ion binding	+1.51	0.0247
47	BM171804	Dr.23461	stc2	Stanniocalcin 2	Hormone activity	+1.52	0.0386
56	AW566602	Dr.36369	olfml3	Olfactomedin-like 3	Extracellular space	+1.62	0.0392
82	BG727594	Dr.12251	cmg2a	Capillary morphogenesis protein 2A	Receptor activity; Protein binding	+1.47	0.0428
96	AI877586	Dr.36948	NRN1	Neuritin	Axonogenesis	+2.19	0.0474
101	BE605771	Dr.9651	Ifitm1	Interferon induced transmembrane protein 1	Anterior/posterior pattern formation	+1.42	0.0507
106	BI882405	Dr.10079	crybb3	Crystallin, beta B3	Structural constituent of eye lens	+1.48	0.0558
206	BG985499	Dr.1940	Spna2	Spectrin alpha 2	Barbed-end actin filament capping	+1.28	0.0752
225	BI673745	Dr.14543	rtdr1	Rhabdoid tumor deletion region protein 1	Binding	+2.03	0.0792
291	AW281815	Dr.22797	LAPTM5	Lysosomal-associated protein transmembrane 5	Lysosome	+1.64	0.0965
